# 

**Published:** 1849-01

**Authors:** 


### CONTENTS.

### PART I.—ORIGINAL COMMUNICATIONS.

ART. 1. Sanguinaria Canadensis as a Therapeutic Agent. By J. L. Mothershead, M. D. - - - - - - 369
ART. 2. Remarks upon Puerperal Fever Complicated with Malaria. By Edward R. Parks, M. D., of Leesburg, Indiana - - - - - - - 374
ART. 3. Rheumatic Affections of the Stomach. By R. R. Stone, M. D., of Solon, Ill. - - - - - - 379
ART. 4. Autopsy of a case of Abscess of the Liver. Reported by Halsey Rosenkrans, M. D., of Ottawa, Ill. 381
ART. 5. Diseases of the West—their Complications, &c. By W. Matthews, M. D., of Eberle, Ind. - - - - 382
ART. 6. A Case of Strangulated Inguinal Hernia mistaken for Bilious Colic by a Thompsonian--Consequent Death. By R. Horsely, medical student - - - - - - 386
ART. 7. Report of a Case of Inflammation of the Fauces and Glottis, resulting in Complete Obstruction of Respiration; Tracheotomy successfully performed. By J. B. Herrick, M. D., Demonstrator of Anatomy in Rush Medical College - - - - 387
ART. 8. Report of a Case of Polypus Uteri. By Dr. R. C. Hamill, of Bloomington, Ind. - - - - 390
ART. 9. Letter from Willi[illegible] G. Edwards, M. D., to Prof. Brainard - - - 393
ART. 10. Iodine an Antidote to the Venom of the Rattle-Snake. By James Whitmire, M. D., of Metamora, Ill. - - - - - - - - 396

### PART II.—REVIEWS AND NOTICES OF NEW WORKS.

ART. 1. The Nature and Treatment of Deafness, and Diseases of the Ear, &c. By William Dufton, M. R. C. S. 399
ART. 2. An Inquiry into the Degree of certainty in Medicine, &c. By Elisha Bartlett, M. D., &c. - - - - 408
ART. 3. A Dispensatory, &c. By Robert Christisen, M. D,. V. P. R. S. E., &c. 413
ART. 4. Lectures on the Theory and Practice of Physic. By Jno. Bell, M. D., &c.; and by William Stokes, M.D., &c. - - - - - - - - 415
ART. 5. On the Cause and treatment of Abortion and Sterility, &c. By James Whitehead, F. R. C. S., &c. 416
ART. 6. The Principles and Practice of Modern Surgery. By Robert Druitt, F. R. C. S. Edited by F. W. Sargent, M. D., &c. - - - - - - 416
ART. 7. A Dictionary of Medical Science, &c. By Robley Dunglison, M. D., &c. - - - - - - 417
ART. 8. Transactions of the American Medical Association. Instituted 1847 417
ART. 9. An Account of some of the most important Diseases peculiar to Women. By Robert Gooch, M. D. - 418
ART. 10. Memoranda on Anatomy, Surgery, and Physiology. By Mark Noble Bower, Surgeon. - - - 418

### PART III.—SELECTIONS.

ART. 1. On the treatment of Cholera 520
ART. 2. On the Semeiology of the Tongue. By Samuel Wright, M. D., of Birmington - - - - - 436
ART. 3. Influence of Quinine on the Spleen in Ague - - - - - 428
ART. 4. Statistics of Amputations in the New York Hospital, from January 1st, 1839, to January 1st, 1848. By Henry W. Buel, M. D., Resident Surgeon - - - - - - - 430
ART. 5. Clinical Lecture on Aphonia and Stammering. By Charles A. Lee, M. D. Delivered before the Medical Class of Geneva College - - - 436
ART. 6. Intermittent Fevers - - 446
ART. 7. Ancient Monuments of the Mississippi Valley. - - - - 447
ART. 8. Can a Reptile live in the Stomach? - - - - - - 447
ART. 9. Kreosote in Erysipelas. By P. Fahnestock, M. D., of Pittsburg 449
ART. 10. On an Electrical Phenomenon observed in Cholera, By J. C. Atkinson, Esq., M. R. C. S., &c., Westminster - - - - - - - 449
ART. 11. New Operation for the Radical Cure of Varicocele. By S. D. Gross, Professor of Surgery in the Medical Department of the University of Louisville - - - - - - 450

### PART IV.—EDITORIALS.

ART. 1. The Cholera - - - - 452

ART. 2. Kosciusko County Medical Society - - - - - - - 453

[illegible] of Western Medical [illegible]

ART. 4. Monster, with a Head resembling a Dog's - - - - - - - 454

ART. 5. Sectional Medicine - - - 457

ART. 6. Miscellaneous Medical Intelli- [illegible]

